# The rise of 3D bioprinting advancements in modeling neurodegenerative diseases

**DOI:** 10.1002/ibra.12196

**Published:** 2025-04-22

**Authors:** Lucia Iafrate, Gianluca Cidonio

**Affiliations:** ^1^ Center for Life Nano‐ & Neuro‐Science (CLN2S) Italian Institute of Technology Rome Italy; ^2^ Department of Mechanical and Aerospace Engineering (DIMA) Sapienza University of Rome Rome Italy

**Keywords:** 3D bioprinting, biofabrication, in vitro models, neurodegenerative diseases, tissue engineering

## Abstract

Neurodegenerative diseases (NDs) are disorders that drastically alter the physiological functioning of neurons in the brain. These processes are often accompanied by abnormal protein aggregates that alter the physical and chemical properties of brain tissue and peripheral nerves. The causes of NDs are complex, involving genetic factors, neuroinflammation, oxidative stress, environmental influences, and lifestyle, while symptoms and progression vary significantly based on the mechanisms of cell death. Currently, no definitive treatment exists for NDs, as the underlying degenerative processes remain poorly understood. Existing therapies focus on symptom alleviation but are insufficient to halt or prevent disease progression. This highlights the urgent need for strategies that mimic the pathophysiology of NDs, facilitating deeper insights and the development of effective treatments. Conventional in vitro and in vivo models attempt to replicate NDs but often fail to capture the physiological complexity of nervous tissue and its interactions. In this context, 3D microfluidic bioprinting emerges as a transformative technology. By enabling precise deposition of cells and biomaterials, it allows the creation of in vitro models with a high degree of structural and functional complexity. These advancements provide a valuable platform for faithfully modeling NDs, bridging critical gaps in our understanding, and paving the way toward innovative therapeutic approaches.

## Graphical abstract

Modeling neurodegenerative diseases: 3D bioprinting as a strategy for replicating the complexity of brain architecture. Recent findings have demonstrated that 3D bioprinting platforms can be exploited to recreate the complex neural pattern, giving the possibility of controlling cell density and scaffold stiffness. This could potentially open new avenues for resembling the diseased neural tissue for the screening of therapeutics towards the treatment of neurodegenerative diseases.

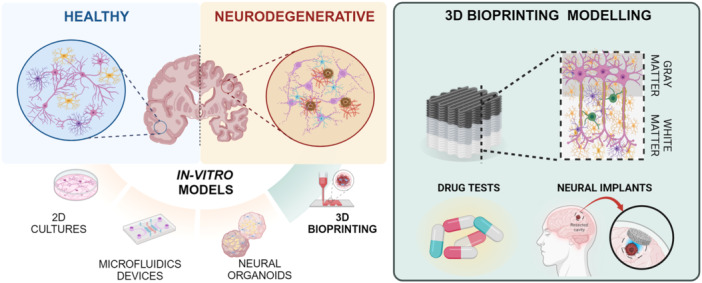

## 1 A NEED FOR NEURODEGENERATIVE DISEASE MODELING

Neurodegenerative diseases (NDs) are disorders characterized by progressive neuronal dysfunction and cell death, often driven by complex interactions involving environmental and genetic factors, oxidative stress, and neuroinflammation.^1^, ^2^, ^3^, ^4^ These processes are frequently accompanied by the accumulation of pathological protein aggregates, which disrupt the structural and functional integrity of the brain and peripheral nervous system.^1^ Common examples include Alzheimer's, Parkinson's, and other tauopathies and synucleinopathies, for which current therapies provide only symptomatic relief, failing to halt or reverse disease progression.^5^ The lack of effective treatments highlights the critical need to develop innovative platforms that replicate the key features of NDs in a controlled environment.^6^, ^7^, ^8^ Such platforms must capture the hierarchical organization and dynamic interactions of brain tissue, providing a foundation to study pathophysiological mechanisms and to improve NDs treatments.

Disease modeling is the results of the synergistic effort from tissue engineering, developmental biology, and biomaterial science to replicate and study pathological mechanisms. Currently, the modeling of NDs poses an enormous challenge.^9^ A primary challenge rests in the possibility to accurately replicate the hierarchical microenvironment of brain tissue and cellular maturation, which are key elements for building realistic in vitro models.^10^ The inherent complexity of brain tissue and the intricate cellular communication are currently limiting the possibility to ultimately and fully capturing the pathophysiology of ND.^10^

**FIGURE 1:**
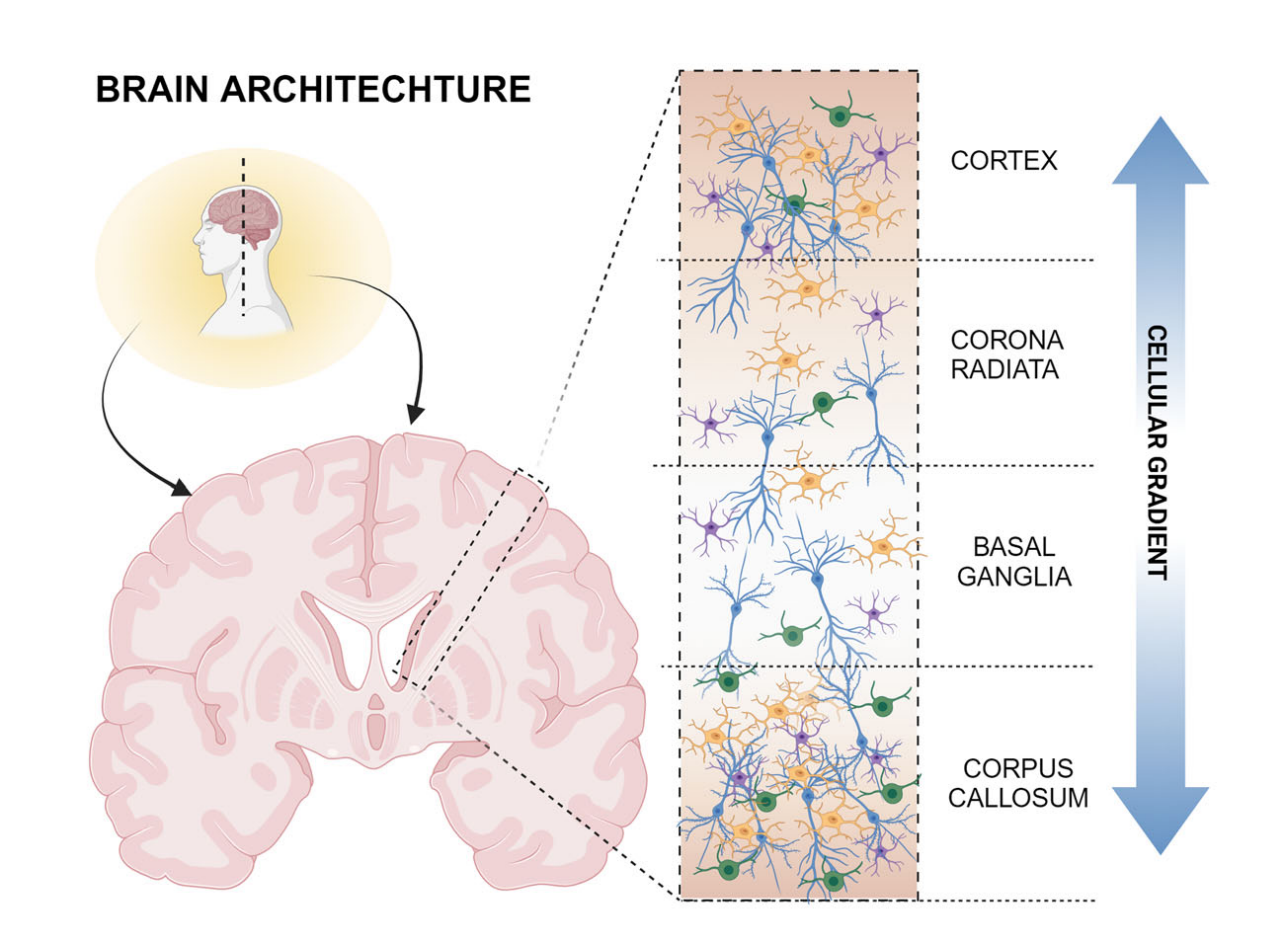
Cellular distribution in brain tissue. Cell density and tissue stiffness depend on brain plasticity and pathological degenerating conditions. [Color figure can be viewed at wileyonlinelibrary.com]

Cellular density and morphology with associated functionality, are found to vary throughout the brain tissue in spatial compartments (Figure 1). Indeed, the white matter hosts myelinated axons, oligodendrocytes, and fibrous astrocytes, while the gray matter houses neuronal cells bodies that form synapses supported by astrocytes and microglia.^11^, ^12^ A crucial role is played by the blood‐brain barrier (BBB), a semi‐permeable barrier that encloses the microvasculature of the central nervous system,^13^ which is formed by endothelial cells that interact with pericytes, astrocytes, and microglia, regulating nutrient passage.^13^, ^14^ The BBB is an essential defense mechanism that protects the brain from toxins and pathogens.^13^ Furthermore, the BBB system has proved to play a significant role in the progression of a number of NDs with inherit neuroinflammation, with associated blocking potential—preventing more than 98% of drugs from entering the nervous system—which impede the direct deliver therapeutics to deep areas of the brain.^13^, ^14^, ^15^ To empower NDs modeling and drug development, the design of three‐dimensional models that mimic the BBB is essential.^16^

In addition to the BBB, the glymphatic system (GS) is increasingly recognized as an essential regulator of brain homeostasis, with its dysfunction closely linked to the development of several NDs.^17^ This glial cell‐driven system plays a vital role in clearing metabolic waste from the brain's interstitial fluid primarily during sleep by using perivascular spaces and aquaporin‐4 (AQP4) water channels located on astrocytic end feet to mediate the exchange of cerebrospinal fluid (CSF) and interstitial fluid.^18^, ^19^ Impairments in glymphatic function can lead to a significant buildup of proteins and waste products, potentially triggering inflammatory responses, features linked to the progression of NDs such as Alzheimer's and Parkinson's diseases. As these disorders advance, they contribute to an increased inflammatory state, creating a vicious cycle that worsens the overall pathology.^18^ Given its significant role in neurodegeneration, incorporating glymphatic dynamics into 3D in vitro models could provide critical insights into waste clearance mechanisms and the role of the inflammation in NDs; however, replicating the dynamics CSF and the clearance of metabolic waste remains challenging, as GS is still not fully understood.^20^

Traditionally, neurodegeneration research has relied on post‐mortem brain samples or longitudinal clinical studies, but they fail to investigate the initial stages of NDs, providing a poorly reliable platform for preclinical studies.^21^ To date, research on NDs has increasingly focused on the exploitation of in vivo animal models.^22^ While these models are useful, their significant physiological differences from humans pose challenges in developing effective therapies.^22^, ^23^ Static in vitro cellular culture models offer the opportunity to investigate the disease progression harnessing cells from patients, providing a human‐specific alternative. However, 2D models are still not able to fully recapitulate the complexity of NDs microenvironment, with the associated impediment for therapeutic testing.^22^, ^24^, ^25^ Among NDs modeling platforms, microfluidic devices offer a more complex environment compared to 2D models, enabling the growth of various cell types in interconnected compartments to better recapitulate the diseased milieu.^23^, ^26^ However, challenges remain in achieving long‐term cell maturation and fully integrating a functional vasculature.^27^ Moreover, microfluidics devices cannot mimic the physiological structure of extracellular matrix (ECM) in vivo, since a 2D monolayer cell culture is often reproduced.^24^

Advancing beyond these limitations, brain organoids have demonstrated the ability to replicate key mechanisms of mammalian neurodevelopment and exhibit characteristics similar to the human brain.^28^ Brain organoids recreate the organization and interaction of different cell types, supporting cell division, ECM production, and the development of morphologies and gene expressions that closely resemble those of the human brain.^21^, ^29^ During the development of brain organoids, careful consideration of the mechanical properties of supporting matrices is crucial, as these parameters significantly influence not only the dimensional growth of the organoids but also their capacity to generate specific neural cell populations. For instance, matrices with intermediate stiffness (*E* = 2–4 kPa) promote dorsal‐ventral development, whereas stiffer matrices (*E* = 12 kPa) restrict growth and rosette formation while encouraging neuronal maturation. In contrast, overly soft matrices (*E* = 0.5 kPa) fail to support proper cellular organization.^30^ However, organoids typically lack a vascular system, which restricts functional growth, facilitating the development of necrotic core.^21^ Finally, mature brain organoids still exhibit embryonic markers, resulting in an incomplete tissue maturation.^31^ At the same time, organoids may undergo neuronal senescence, caused by both genetic factors and long‐term culture. Altogether, these features are limiting the use of brain organoids as reliable models for human brain aging and NDs.^21^, ^32^ Thus, the possibility to replicate the intricate architectural complexity of the brain with an in vitro model still remains unsolved. Particularly, the current inability to closely replicate the spatial arrangements of cells, biological substances, and materials, following a gradient‐like architecture with a long‐term stability, is failing to imitate NDs, thus slowing the fabrication of safe and efficacious drug screening platform.

## 2 3D BIOPRINTING AS A DISEASE MODELING PLATFORM

Today, 3D bioprinting is gaining increasing attention as a powerful platform for disease modeling, moving beyond dated 2D culture systems.^33^ 3D bioprinting enables the precise deposition of cells and materials, creating complex tissue models that replicate in vivo cellular architecture and interactions.^10^ This level of complexity is challenging to achieve with other methods, making 3D models valuable for studying diseases, including NDs.^10^ The 3D bioprinting process provides the use of bioinks (cells mixed with biomaterial inks), which can be enriched with bioactive molecules to enhance cell survival, differentiation, and functionality for long‐term stability.^24^ The extrusion of cell‐laden inks is associated with a range of shear stress imposed on flowing cells, which may affect differentiation and survival. To preserve cellular integrity and ultimate functionality, a bioink with shear‐thinning properties can be designed to dissipate stresses during deposition.^34^, ^35^, ^36^ At the same time, to replicate neural tissues, constructs must be engineered with Young's moduli that can closely match the brain mechanical properties (2–4 kPa).^37^ However, mechanical competencies for NDs are lost with lower Young's moduli for diseases such as Alzheimer's, Parkinson's, and amyotrophic lateral sclerosis.^38^, ^39^ The use of low‐viscosity bioinks allows the creation of a niche to recapitulate cell‐ECM interactions in both healthy and pathological conditions, while resulting limits in preserving models for long‐term culture due to the elevated degradation rate and poor in vitro stability.^37^

A number of 3D bioprinted models have been engineered to resemble the nervous tissue. Lozano and colleagues demonstrated the fabrication of a 3D bioprinted layered construct imitating the architecture of the human cortex, reporting consistent survival and differentiation of neural cells.^34^, ^40^, ^41^ A mini‐neural tissue was created by Gu and colleagues by using human cortical neural cells in a polysaccharide hydrogel, exhibiting good viability and differentiation, but failing to fully replicate the complex neuronal microenvironment.^22^, ^42^, ^43^ Hinton and co‐workers proposed the assembly of human brain structures by freeform reversible embedding of suspended hydrogels method and magnetic resonance imaging, producing a scaffold with the complex anatomical brain features but failing to maintain the viability of neurons in the inner structure.^44^ Despite that, this study demonstrated the potential of 3D bioprinting approaches to assemble brain‐like tissues, and superior details, such as cell subtypes and vascularization, may be needed for more advanced applications.^41^, ^44^ Finally, the incorporation of GS is another critical frontier in 3D bioprinting for brain modeling. The inclusion of GS in 3D bioprinted models adds layers of complexity, as accurately replicating perivascular spaces and dynamic CSF flow. However, it remains technically demanding.^45^ Furthermore, astrocyte maturation and stable AQP4 expression in vitro are difficult to achieve, potentially limiting the functionality of glymphatic‐like scaffolds.^46^, ^47^ Altogether, 3D bioprinting can offer an advanced tool in disease modeling, providing new opportunities to understand disease mechanisms and develop personalized therapies.

## 3 BENEFITS AND CHALLENGES IN REPLICATING NEURODEGENERATIVE PATHOLOGIES VIA 3D BIOPRINTING

**FIGURE 2:**
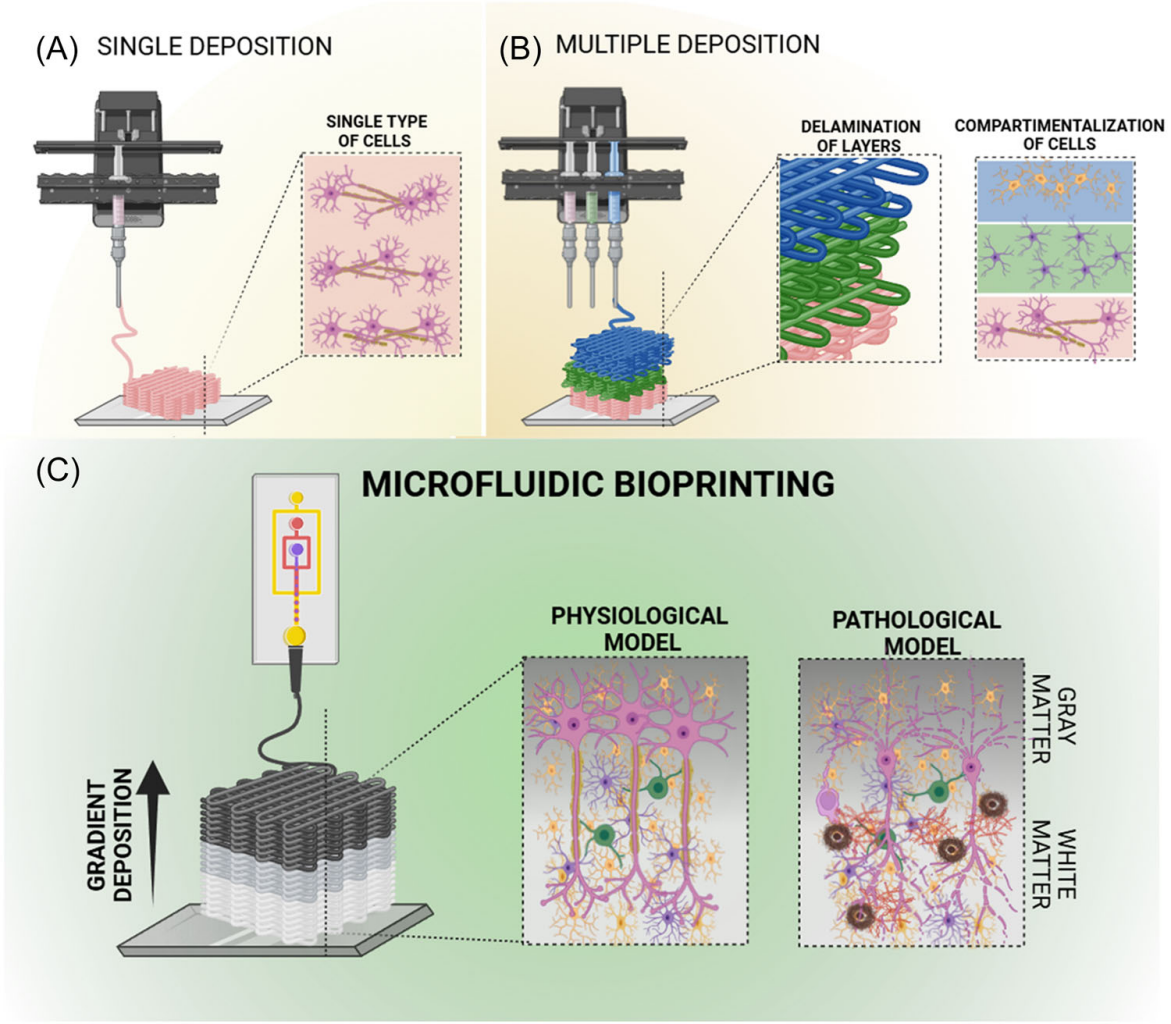
Comparison of three bioprinting strategies. Bioprinting strategies include: (A) Single deposition: scaffolds with high resolution but limited in mimicking the physiological cell density. (B) Multiple deposition: by combining different materials, it is possible to achieve a multicellular scaffold, but it is limited in the interpenetration between different cell types. Furthermore, delamination, caused by the loss of cohesion between the various bioinks, can reduce resolution. (C) Microfluidic‐assisted 3D bioprinting: a promising strategy to recreate both physiological and pathological conditions by controlling cellular and materials densities. [Color figure can be viewed at wileyonlinelibrary.com]

The use of 3D bioprinting to model NDs offers substantial benefits but also presents considerable challenges.^48^ With 3D bioprinting, precise control over the positioning of cells, biomaterials, and growth factors is possible, allowing the development of models that closely resemble the natural brain environment.^10^, ^43^, ^49^ Furthermore, bioactive inks engineered for 3D bioprinting purposes, typically demonstrate the ability to promote cell adhesion and growth, which are essential features for the development of functional nervous system.^50^ However, the use of a single printhead with a unique cell type loaded into a bioink during 3D bioprinting processes might not be sufficient to recreate the structure of brain tissue, as it does not allow for the replication of its complex multicellular structure, particularly in terms of cellular density control (Figure 2A).

To overcome such limitations, multiple printhead 3D bioprinting can be employed to deposit different cell types by the means of independent printheads and bioinks to match the neural tissue complexity and heterogeneity.^10^, ^50^ The use of a multi‐printheads 3D bioprinter would favor the assembly of diverse cellular populations and biochemical patterns, to resemble the hierarchical arrangement of the brain tissue (Figure 2B). Nevertheless, multi‐printhead 3D bioprinting has not yet achieved the fabrication of a physiological model due to delamination issues and a lack of control over the arrangement of cellular and material densities in 3D to foster cell‐to‐cell communication. Thus, there is a need for new technological advancements for the 3D fabrication of disease brain tissue that might rescue the hierarchical patterning of cells, biologics, and materials to recapitulate the altered microenvironment.

## 4 THE UNTAPPED POTENTIAL FOR HIERARCHICAL BIOFABRICATION OF A BIOMIMETIC NDS PLATFORM

Recently, microfluidic‐assisted 3D bioprinting has emerged as a promising method in biofabrication, allowing for the engineering of complex structures with high resolution accommodating a wide range of cellular components and bioink viscosities.^51^, ^52^ Harnessing the geometry of microfluidic printheads, it is possible to print physiological patterns by exploiting gradient spatial transitions between bioinks, resulting in structures with well‐defined properties. We have recently demonstrated the possibility to integrate a microfluidic logic with a 3D bioprinting platform, offering a new approach to create 3D gradients of discrete compartments (e.g., porosities, biologics) by adjusting the bioink flow rates while finely tuning the dispensing,^52^, ^53^, ^54^, ^55^, ^56^ as well as patterning biomaterials^56^ and delivering compartmentalization in three‐dimensional tissue models.^57^ In a further attempt, Serex and colleagues demonstrated that by alternating bioinks with different Young's moduli via microfluidic dispensing, a patterned library of mechanical properties can be delivered in 3D.^52^, ^58^ Building on these results, it is clear that microfluidic‐assisted 3D bioprinting approaches offer numerous opportunities to increase the complexity of printed constructs by controlling the deposition of both biomaterials and cells, modulating cell density and controlling material stiffness, features of great interest in creating brain models.^51^ Indeed, the 3D bioprinting of gradient‐patterned neurons and glial cells would sustain an increasingly functional response compared to spatially unresolved cellular deposition (Figure 2C). Thus, we now have the opportunity to fabricate NDs models that would recapitulate pathological cellular density, influencing interactions, development, and, ultimately, survival.^59^ Since the microstructure of brain tissue has not only spatially but also temporally changes depending on both neural plasticity and pathological conditions, the ability to modulate the stiffness of 3D bioprinted constructs offers a valuable advantage in modeling ND.^11^, ^60^ In fact, the ability to print different materials in a controlled manner allows for the modulation of stiffness in specific areas, either by leveraging the unique mechanical properties of the bioinks or by adjusting their degradation kinetics to create softer regions within the scaffold. This presents a significant advantage in recreating brain tissue, as single deposition bioprinting cannot achieve this. Additionally, without the use of microfluidic printheads, modulating degradation kinetics in multi‐printhead 3D bioprinting could increase the risk of scaffold delamination. Despite the potential of 3D microfluidic‐assisted bioprinting, there are limitations, such as the long‐term stability of 3D bioprinted constructs due to the softness of the neuro‐bioinks used for tissue assembly.^51^ Indeed, one of the major challenges in fabricating 3D neural tissue is the development of functional bioinks that simultaneously possess printability, rapid gelation, and compatibility properties to accommodate neural cell culture.^42^ To date, a number of printable bioinks have been found unable to support neurons viability and functionality following 3D deposition. Hydrogels such as collagen, Matrigel, hyaluronic acid, and laminin, while offering good biocompatibility with neural cells, lack the necessary printability properties and must be combined with other printable polymers to augment the deposition stability while maintaining the desired neural functionality support.^60^ Additionally, soft bioinks have demonstrated limited in vitro stability over the long term due to their relatively fast degradation rates compared to the culture periods needed for studying ND, restricting their use in long‐term culture and studies.^61^ Future effort in optimizing bioinks design, especially for microfluidic‐assisted 3D bioprinting platforms, might aid the fabrication of biomimetic NDs conditions, advancing the testing of revolutionary treatments on models that better recapitulate the patient‐specific physiology and disease state.

## 5 OPEN QUESTIONS AND FUTURE PERSPECTIVES

Significant strides have been made in NDs modeling, yet replicating the intricate microenvironment, cellular architecture, and dynamic processes of the human brain remains a challenge.^9^, ^10^ Current platforms, such as traditional 2D cultures and static 3D models, have provided valuable insights but fail to fully capture the multifaceted nature of NDs. These models often lack the hierarchical organization, cellular diversity, and dynamic interactions essential for studying disease mechanisms and testing therapeutics.^21^, ^22^, ^23^, ^24^, ^25^, ^27^, ^31^, ^32^

Technological limitations significantly hinder the development of effective therapies for NDs. One of the main challenges is the fabrication of models with sufficient long‐term stability and hierarchical organization, which are critical for generating neural tissues suitable for studying disease progression.^37^, ^42^, ^43^ To date, preclinical research relies on animal or ex vivo models.^22^ However, these approaches present ethical concerns and raise questions about the reliability of obtained data, making them nonideal solutions for NDs modeling.

3D bioprinting platforms, and specifically microfluidic‐assisted bioprinters, hold a great potential to overcome these limitations, enabling the precise spatial arrangement of cells, biomaterials, and signaling molecules, offering the possibility to closely replicate the complexity of brain cell density.^10^, ^51^, ^52^ Particularly, the multi‐printhead systems provided by microfluidic logic allow for the creation of constructs with controlled stiffness, cellular density, and biochemical environments, leveraging the extrusion of gradient‐patterned bioinks.^55^, ^56^, ^57^, ^58^ This technology could lead to more accurate models of ND pathophysiology, particularly if used to fabricate patient‐specific brain tissue constructs.

Indeed, each patient exhibits unique physiological results of NDs, meaning therapies may not be equally effective for all individuals. The fabrication of patient‐specific models would significantly enhance personalized approaches to NDs treatment. For example, induced pluripotent stem cells can be engineered from fibroblasts isolated from specific patients, enabling large‐scale production of neural cells affected by the respective ND.^62^ By integrating brain imaging data, it would be possible to design a custom bioprinted model that accurately replicate the pathological cellular densities of the patient within brain tissue.

Furthermore, with the support of advanced research into novel functionalization processes for neuro‐biomaterials, more stability could be provided to NDs models and, by involving the microfluidic‐assisted 3D bioprinting, a cellular hierarchical architecture could be reached providing essential insights in NDs modeling.

In an increasingly futuristic endeavor, patient‐specific 3D bioprinted constructs could be involved to slow the ND progression. However, significant challenges must still be overcome. The long‐term stability and maturation of 3D bioprinted constructs are limited due to the inadequate mechanical properties of current neural bioinks, which struggle to maintain structural integrity during extended culture periods.^37^ Additionally, replicating functional vascularization and glymphatic‐like features in these models remains a significant hurdle, hindering the replication of critical physiological processes such as nutrient transport, waste clearance, and inflammation regulation.^27^, ^45^

Altogether, 3D bioprinting platforms offer promising scenarios for the future. The ability to create in vitro models with specific patterns to study NDs represents a significant step forward. Further advancements in technical and engineering innovation will be crucial for accelerating the development of functional neural tissues. Despite current limitations, 3D bioprinting remains one of the most promising technological pathways for understanding ND mechanisms and advancing personalized, targeted clinical therapies.

## AUTHOR CONTRIBUTIONS

Lucia Iafrate wrote and edited the manuscript and prepared the figures. Gianluca Cidonio conceptualized, supervised, acquired funding, wrote, and/or edited the manuscript before submission.

## CONFLICT OF INTEREST STATEMENT

The authors declare no conflicts of interest.

## ETHICS STATEMENT

Not applicable.

## Data Availability

The manuscript does not contain novel data, and all the information provided can be sourced from literature.

## References

[1] Evangelisti C , Ramadan S , Orlacchio A , Panza E . Experimental cell models for investigating neurodegenerative diseases. Int J Mol Sci. 2024;25(17):9747. 10.3390/ijms25179747 39273694 PMC11396244

[2] Samanta S , Chakraborty S , Bagchi D . Pathogenesis of neurodegenerative diseases and the protective role of natural bioactive components. J Am Nutr Assoc. 2024;43(1):20‐32. 10.1080/27697061.2023.2203235 37186678

[3] Cooper O , Hallett P , Isacson O . Upstream lipid and metabolic systems are potential causes of Alzheimer's disease, Parkinson's disease and dementias. FEBS J. 2024;291(4):632‐645. 10.1111/febs.16638 36165619 PMC10040476

[4] Scarian E , Viola C , Dragoni F , et al. New insights into oxidative stress and inflammatory response in neurodegenerative diseases. Int J Mol Sci. 2024;25(5):2698. 10.3390/ijms25052698 38473944 PMC10931808

[5] Ricci C . Neurodegenerative disease: from molecular basis to therapy. Int J Mol Sci. 2024;25(2):967. 10.3390/ijms25020967 38256040 PMC10815646

[6] Wei M , Yang Z , Li S , Le W . Nanotherapeutic and stem cell therapeutic strategies in neurodegenerative diseases: a promising therapeutic approach. Int J Nanomedicine. 2023;18:611‐626. 10.2147/IJN.S395010 36760756 PMC9904216

[7] Van Schependom J , D'haeseleer M . Advances in neurodegenerative diseases. J Clin Med. 2023;12(5):1709. 10.3390/jcm12051709 36902495 PMC10002914

[8] Lamptey RNL , Chaulagain B , Trivedi R , Gothwal A , Layek B , Singh J . A review of the common neurodegenerative disorders: current therapeutic approaches and the potential role of nanotherapeutics. Int J Mol Sci. 2022;23(3):1851. 10.3390/ijms23031851 35163773 PMC8837071

[9] Rey F , Barzaghini B , Nardini A , et al. Advances in tissue engineering and innovative fabrication techniques for 3‐D‐structures: translational applications in neurodegenerative diseases. Cells. 2020;9(7):1636. 10.3390/cells9071636 32646008 PMC7407518

[10] Pereira I , Lopez‐Martinez MJ , Samitier J . Advances in current in vitro models on neurodegenerative diseases. Front Bioeng Biotechnol. 2023;11(November):1‐20. 10.3389/fbioe.2023.1260397 PMC10658011 38026882

[11] Budday S , Ovaert TC , Holzapfel GA , Steinmann P , Kuhl E . Fifty Shades of Brain: A Review on the Mechanical Testing and Modeling of Brain Tissue. 27. Springer; 2020. 10.1007/s11831-019-09352-w

[12] Budday S , Steinmann P , Kuhl E . Physical biology of human brain development. Front Cell Neurosci. 2015;9(July):1‐17. 10.3389/fncel.2015.00257 26217183 PMC4495345

[13] Wu D , Chen Q , Chen X , Han F , Chen Z , Wang Y . The blood–brain barrier: structure, regulation, and drug delivery. Signal Transduct Target Ther. 2023;8(1):217. 10.1038/s41392-023-01481-w 37231000 PMC10212980

[14] Kadry H , Noorani B , Cucullo L . A blood‐brain barrier overview on structure, function, impairment, and biomarkers of integrity. Fluids Barriers CNS. 2020;17(1):69. 10.1186/s12987-020-00230-3 33208141 PMC7672931

[15] Akhtar A , Andleeb A , Waris TS , et al. Neurodegenerative diseases and effective drug delivery: a review of challenges and novel therapeutics. J Controlled Release. 2021;330:1152‐1167. 10.1016/j.jconrel.2020.11.021 33197487

[16] Slanzi A , Iannoto G , Rossi B , Zenaro E , Constantin G . In vitro models of neurodegenerative diseases. Front Cell Dev Biol. 2020;8(May):328. 10.3389/fcell.2020.00328. 32528949 PMC7247860

[17] Verghese JP , Terry A , de Natale ER , Politis M . Research evidence of the role of the glymphatic system and its potential pharmacological modulation in neurodegenerative diseases. J Clin Med. 2022;11(23):6964. 10.3390/jcm11236964 36498538 PMC9735716

[18] Cai Y , Zhang Y , Leng S , et al. The relationship between inflammation, impaired glymphatic system, and neurodegenerative disorders: a vicious cycle. Neurobiol Dis. 2024;192(January):106426. 10.1016/j.nbd.2024.106426 38331353

[19] Buccellato FR , D'Anca M , Serpente M , Arighi A , Galimberti D . The role of glymphatic system in Alzheimer's and Parkinson's disease pathogenesis. Biomedicines. 2022;10(9):2261. 10.3390/biomedicines10092261 36140362 PMC9496080

[20] Spitz S , Ko E , Ertl P , Kamm RD . How organ‐on‐a‐chip technology can assist in studying the role of the glymphatic system in neurodegenerative diseases. Int J Mol Sci. 2023;24(3):2171. 10.3390/ijms24032171 36768495 PMC9916687

[21] Urrestizala‐Arenaza N , Cerchio S , Cavaliere F , Magliaro C . Limitations of human brain organoids to study neurodegenerative diseases: a manual to survive. Front Cell Neurosci. 2024;18(July):1419526. 10.3389/fncel.2024.1419526. 39049825 PMC11267621

[22] Iafrate L , Benedetti MC , Donsante S , et al. Modelling skeletal pain harnessing tissue engineering. In vitro models. 2022;1(4‐5):289‐307. 10.1007/s44164-022-00028-7 36567849 PMC9766883

[23] Miny L , Maisonneuve BGC , Quadrio I , Honegger T . Modeling neurodegenerative diseases using in vitro compartmentalized microfluidic devices. Front Bioeng Biotechnol. 2022;10(June):1‐17. 10.3389/fbioe.2022.919646 PMC9263267 35813998

[24] de Vitis E , Stanzione A , Romano A , et al. The evolution of technology-driven in vitro models for neurodegenerative diseases. Adv Sci. 2024;11(16):2304989. 10.1002/advs.202304989. PMC11040362 38366798

[25] Centeno EGZ , Cimarosti H , Bithell A . 2D versus 3D human induced pluripotent stem cell‐derived cultures for neurodegenerative disease modelling. Mol Neurodegener. 2018;13(1):27. 10.1186/s13024-018-0258-4 29788997 PMC5964712

[26] Amartumur S , Nguyen H , Huynh T , et al. Neuropathogenesis‐on‐chips for neurodegenerative diseases. Nat Commun. 2024;15(1):2219. 10.1038/s41467-024-46554-8 38472255 PMC10933492

[27] Akcay G , Luttge R . Microenvironments matter: advances in Brain‐on‐Chip. Biosensors. 2023;13(5):551. 10.3390/bios13050551 37232912 PMC10216565

[28] Chiaradia I , Lancaster MA . Brain organoids for the study of human neurobiology at the interface of in vitro and in vivo. Nature Neurosci. 2020;23(12):1496‐1508. 10.1038/s41593-020-00730-3 33139941

[29] Tekin H , Simmons S , Cummings B , et al. Effects of 3D culturing conditions on the transcriptomic profile of stem‐cell‐derived neurons. Nat Biomed Eng. 2018;2(7):540‐554. 10.1038/s41551-018-0219-9 30271673 PMC6157920

[30] Li M , Sun H , Hou Z , Hao S , Jin L , Wang B . Engineering the physical microenvironment into neural organoids for neurogenesis and neurodevelopment. Small. 2024;20(6):1‐20. 10.1002/smll.202306451 37771182

[31] Andrews MG , Kriegstein AR . Challenges of organoid research. Annu Rev Neurosci. 2022;45:23‐39. 10.1146/annurev-neuro-111020-090812 34985918 PMC10559943

[32] Kelava I , Lancaster MA . Dishing out mini‐brains: current progress and future prospects in brain organoid research. Dev Biol. 2016;420(2):199‐209. 10.1016/j.ydbio.2016.06.037 27402594 PMC5161139

[33] Jiang W , Mei H , Zhao S . Applications of 3D bio‐printing in tissue engineering and biomedicine. J Biomed Nanotechnol. 2021;17(6):989‐1006. 10.1166/jbn.2021.3078 34167615

[34] Cadena M , Ning L , King A , et al. 3D bioprinting of neural tissues. Adv Healthcare Mater. 2021;10(15):2001600. 10.1002/adhm.202001600 PMC8711131 33200587

[35] Blaeser A , Duarte Campos DF , Puster U , Richtering W , Stevens MM , Fischer H . Controlling shear stress in 3D bioprinting is a key factor to balance printing resolution and stem cell integrity. Adv Healthcare Mater. 2016;5(3):326‐333. 10.1002/adhm.201500677 26626828

[36] Cidonio G , Glinka M , Dawson JI , Oreffo ROC . The cell in the ink: improving biofabrication by printing stem cells for skeletal regenerative medicine. Biomaterials. 2019;209(April):10‐24. 10.1016/j.biomaterials.2019.04.009 31022557 PMC6527863

[37] Qiu B , Bessler N , Figler K , et al. Bioprinting neural systems to model central nervous system diseases. Adv Funct Mater. 2020;30(44):1910250. 10.1002/adfm.201910250 34566552 PMC8444304

[38] Budday S , Sommer G , Birkl C , et al. Mechanical characterization of human brain tissue. Acta Biomater. 2017;48:319‐340. 10.1016/j.actbio.2016.10.036 27989920

[39] Feng Y , Murphy MC , Hojo E , Li F , Roberts N . Magnetic resonance elastography in the study of neurodegenerative diseases. J Magn Reson Imaging. 2024;59(1):82‐96. 10.1002/jmri.28747 37084171

[40] Lozano R , Stevens L , Thompson BC , et al. 3D printing of layered brain‐like structures using peptide modified gellan gum substrates. Biomaterials. 2015;67:264‐273. 10.1016/j.biomaterials.2015.07.022 26231917

[41] Joung D , Lavoie NS , Guo SZ , Park SH , Parr AM , McAlpine MC . 3D printed neural regeneration devices. Adv Funct Mater. 2020;30(1):1906237. 10.1002/adfm.201906237. PMC7007064 32038121

[42] Gu Q , Tomaskovic‐Crook E , Wallace GG , Crook JM . 3D bioprinting human induced pluripotent stem cell constructs for in situ cell proliferation and successive multilineage differentiation. Adv Healthcare Mater. 2017;6(17):1700175. 10.1002/adhm.201700175. 28544655

[43] Knowlton S , Cho Y , Li XJ , Khademhosseini A , Tasoglu S . Utilizing stem cells for three‐dimensional neural tissue engineering. Biomater Sci. 2016;4(5):768‐784. 10.1039/c5bm00324e 26890524

[44] Hinton TJ , Jallerat Q , Palchesko RN , et al. Three‐dimensional printing of complex biological structures by freeform reversible embedding of suspended hydrogels. Sci Adv. 2015;1(9):1‐10. 10.1126/sciadv.1500758 PMC4646826 26601312

[45] Nikolakopoulou P , Rauti R , Voulgaris D , Shlomy I , Maoz BM , Herland A . Recent progress in translational engineered in vitro models of the central nervous system. Brain. 2020;143(11):3181‐3213. 10.1093/BRAIN/AWAA268 33020798 PMC7719033

[46] Szu JI , Binder DK . The role of astrocytic aquaporin‐4 in synaptic plasticity and learning and memory. Front Integr Neurosci. 2016;10(2016):1‐16. 10.3389/fnint.2016.00008 26941623 PMC4764708

[47] Silverglate B , Gao X , Lee HP , Maliha P , Grossberg GT . The aquaporin‐4 water channel and updates on its potential as a drug target for Alzheimer's disease. Expert Opin Ther Targets. 2023;27(7):523‐530. 10.1080/14728222.2023.2240017 37475487

[48] D'Antoni C , Mautone L , Sanchini C , et al. Unlocking neural function with 3D in vitro models: a technical review of self‐assembled, guided, and bioprinted brain organoids and their applications in the study of neurodevelopmental and neurodegenerative disorders. Int J Mol Sci. 2023;24(13):10762. 10.3390/ijms241310762 37445940 PMC10341866

[49] Zhu W , O'Brien C , O'Brien JR , Zhang LG . 3D nano/microfabrication techniques and nanobiomaterials for neural tissue regeneration. Nanomedicine. 2014;9(6):859‐875. 10.2217/nnm.14.36 24981651

[50] Knowlton S , Anand S , Shah T , Tasoglu S . Bioprinting for neural tissue engineering. Trends Neurosci. 2018;41(1):31‐46. 10.1016/j.tins.2017.11.001 29223312

[51] du Chatinier DN , Figler KP , Agrawal P , Liu W , Zhang YS . The potential of microfluidics‐enhanced extrusion bioprinting. Biomicrofluidics. 2021;15(4):041304. 10.1063/5.0033280 34367403 PMC8324304

[52] Serpe F , Casciola CM , Ruocco G , Cidonio G , Scognamiglio C . Microfluidic fiber spinning for 3D bioprinting: harnessing microchannels to build macrotissues. Int J Bioprint. 2024;10(1):1404. 10.36922/ijb.1404

[53] Wang M , Li W , Mille LS , et al. Digital light processing based bioprinting with composable gradients. Adv Mater. 2022;34(1):1‐13. 10.1002/adma.202107038 PMC8741743 34609032

[54] Idaszek J , Costantini M , Karlsen TA , et al. 3D bioprinting of hydrogel constructs with cell and material gradients for the regeneration of full‐thickness chondral defect using a microfluidic printing head. Biofabrication. 2019;11(4):044101. 10.1088/1758-5090/ab2622 31151123

[55] Marcotulli M , Tirelli MC , Volpi M , et al. Microfluidic 3D printing of emulsion ink for engineering porous functionally graded materials. Adv. Mater. Technol. 2023;8(5):1‐12. 10.1002/admt.202201244

[56] Mohammadi S , D'Alessandro S , Bini F , Marinozzi F , Cidonio G . Development of a microfluidic‐assisted open‐source 3D bioprinting system (MOS3S) for the engineering of hierarchical tissues. HardwareX. 2024;18:e00527. 10.1016/j.ohx.2024.e00527 38596662 PMC11002860

[57] D'Alessandro S , Mohammadi S , Iafrate L , et al. Hybrid 3D microfluidic bioprinting for the engineering of cancer models and tissue substitutes. Virtual Phys Prototyp. 2024;19(1):e2419411. 10.1080/17452759.2024.2419411

[58] Serex L , Bertsch A , Renaud P . Microfluidics: a new layer of control for extrusion‐based 3D printing. Micromachines. 2018;9(2):86. 10.3390/mi9020086 30393362 PMC6187762

[59] Zhuang P , Sun AX , An J , Chua CK , Chew SY . 3D neural tissue models: from spheroids to bioprinting. Biomaterials. 2018;154:113‐133. 10.1016/j.biomaterials.2017.10.002 29120815

[60] Samanipour R , Tahmooressi H , Rezaei Nejad H , Hirano M , Shin SR , Hoorfar M . A review on 3D printing functional brain model. Biomicrofluidics. 2022;16(1):011501. 10.1063/5.0074631. 35145569 PMC8816519

[61] Chen XB , Fazel Anvari‐Yazdi A , Duan X , et al. Biomaterials/bioinks and extrusion bioprinting. Bioactive Materials. 2023;28:511‐536. 10.1016/j.bioactmat.2023.06.006 37435177 PMC10331419

[62] Dimos JT , Rodolfa KT , Niakan KK , et al. Induced pluripotent stem cells generated from patients with ALS can be differentiated into motor neurons. Science. 2008;321(5893):1218‐1221. 10.1126/science.1158799 18669821

